# Versatile Nanoscale Three-Terminal Memristive Switch Enabled by Gating

**DOI:** 10.1021/acsnano.3c11373

**Published:** 2024-04-09

**Authors:** Mila Lewerenz, Elias Passerini, Bojun Cheng, Markus Fischer, Alexandros Emboras, Mathieu Luisier, Ueli Koch, Juerg Leuthold

**Affiliations:** † TH Zurich, Institute of Electromagnetic Fields (IEF), 8092 Zürich, Switzerland; ‡ The Hong Kong University of Science and Technology, Thrust of Microelectronics, Guangzhou 529200, China; § ETH Zurich, Integrated Systems Laboratory (IIS), 8092 Zürich, Switzerland

**Keywords:** memristor, memristive switching, resistive switching, three-terminal, gating, electrochemical cells

## Abstract

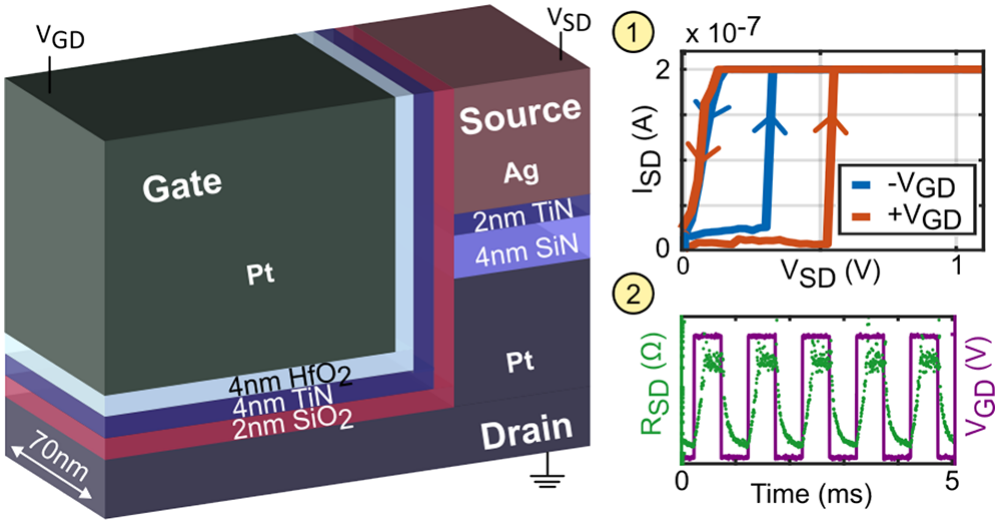

A three-terminal memristor with an ultrasmall footprint of only 0.07 μm^2^ and critical dimensions of 70 nm × 10 nm × 6 nm is introduced. The device’s feature is the presence of a gate contact, which enables two operation modes: either tuning the set voltage or directly inducing a resistance change. In *I*–*V* mode, we demonstrate that by changing the gate voltages between ±1 V one can shift the set voltage by 69%. In pulsing mode, we show that resistance change can be triggered by a gate pulse. Furthermore, we tested the device endurance under a 1 kHz operation. In an experiment with 2.6 million voltage pulses, we found two distinct resistance states. The device response to a pseudorandom bit sequence displays an open eye diagram and a success ratio of 97%. Our results suggest that this device concept is a promising candidate for a variety of applications ranging from Internet-of-Things to neuromorphic computing.

Memristors are devices that encode data in their resistance state.^1^ Two-terminal (2T) memristors have already been demonstrated in various applications, including neuromorphic computing,^2−8^ in-memory computing,^9,10^ and reservoir computing.^11,12^ They offer compact dimensions,^13,14^ low switching energies,^15^ short switching times,^16,17^ and long retention times. However, the potential applications of 2T memristors are restricted, as they require selectors for certain computing operations.^18^ Another challenge is that the read and set operations use the same electronic contacts, which inherently couples the two operations. Lastly, 2T devices often display device-to-device variation in the set voltages, making their integration into crossbar arrays more complex. Such shortcomings can be overcome by introducing a third terminal to a 2T memristor, thus forming a three-terminal (3T) device. The third terminal provides an additional degree of control over the resistance of the device and allows for the implementation of more complex types of functionalities.

State-of-the-art memristive resistance switches are based on different effects, such as ion movement, phase change, ferroelectric, and 2D material effects.^19,20^ One notable example is the electrochemical metallization (ECM) memristor, which relies on metal ion migration and electrochemical reactions, leading to filament formation between two metal electrodes. Various active metals (Ag, Cu, etc.) as well as their alloys^21−23^ have been investigated. The active size can go down to a few dozens of nanometers with the ultimate limit being the displacement of a few or even a single atom.

In the past decade, there has been an increasing interest in the development of 3T memristors that have a third metal electrode.^18,24−41^ State-of-the-art research in the field of 3T memristors has shown promising results with the gate being used to initiate the memristive set and reset,^24−27,29^ suppressing sneak currents^18^ and shifting the set voltage.^28^ Hasegawa et al. demonstrated in 2010 how a gate can be used to induce nucleation, formation, and dissolution of ECM-based devices (Ag and Cu) by using the gate as the source of the active material.^24^ Subsequently, they reduced the gate distance to 20 nm and extended the concept to valence change memories as well.^35^ Similarly, in a silicon memristor/graphene heterojunction barristor, it was shown that the gate can tune the set operation of the device.^28^ Gate voltages of up to 20 V were used to lower the set voltage from 4.7 V down to 1.9 V. Nonetheless, to the best of the authors’ knowledge, so far, the development has focused on demonstrating that 3T devices can be used either to initiate the set/reset operation or to control the set voltage. Combining the two functionalities in a single device and using the gate to either initiate resistive switching or shift the set voltage at low gate voltages on a small footprint have not yet been shown.

In this paper, we present an ECM-based 3T memristor with two different operation modes, one where the additional gate is used to control the set voltage (“set-voltage tuning” mode) and one where it directly modulates the resistance switching (“switching” mode). The proposed device combines an ultracompact Ag-SiO_2_-Pt memristor with a Pt gate. It has an ultrasmall footprint of only 0.07 μm^2^, with an interaction volume of 70 × 10 × 6 nm^3^. We characterize the devices’ current–voltage characteristics first, later called DC IV, and then use a pulsed setup to demonstrate high endurance and low device-to-device variations. Its robustness to arbitrary signals is tested using pulse trains with pseudorandom bit sequences. Our results show a 3T memristor with reliable switching for over 2.6 million voltage pulses and with two operation modes. The “set-voltage-tuning” mode relies on a constant gate offset of both polarities to vary the DC IV switching characteristics of the device. Constant gate voltages of ±1 V shift the set voltage by 69.2% (from 0.55 V to 0.325 V) while keeping the typical memristor hysteresis intact. In the “resistance switching” mode, the gate is used to initiate the resistance change of the central SiO_2_ contact. For this, the bias voltage across source(Ag)–drain(Pt) is kept constant. We show two distinct device resistance distributions for 2.6 million pulses, repeatability for different devices, as well as an open eye diagram for pseudorandom bit sequence inputs. Overall, our 3T memristor offers a promising platform for exploring various paradigms in memory and computation.

## Results/Discussion

### Device Concept and Two-Terminal Characterization

**Figure 1:**
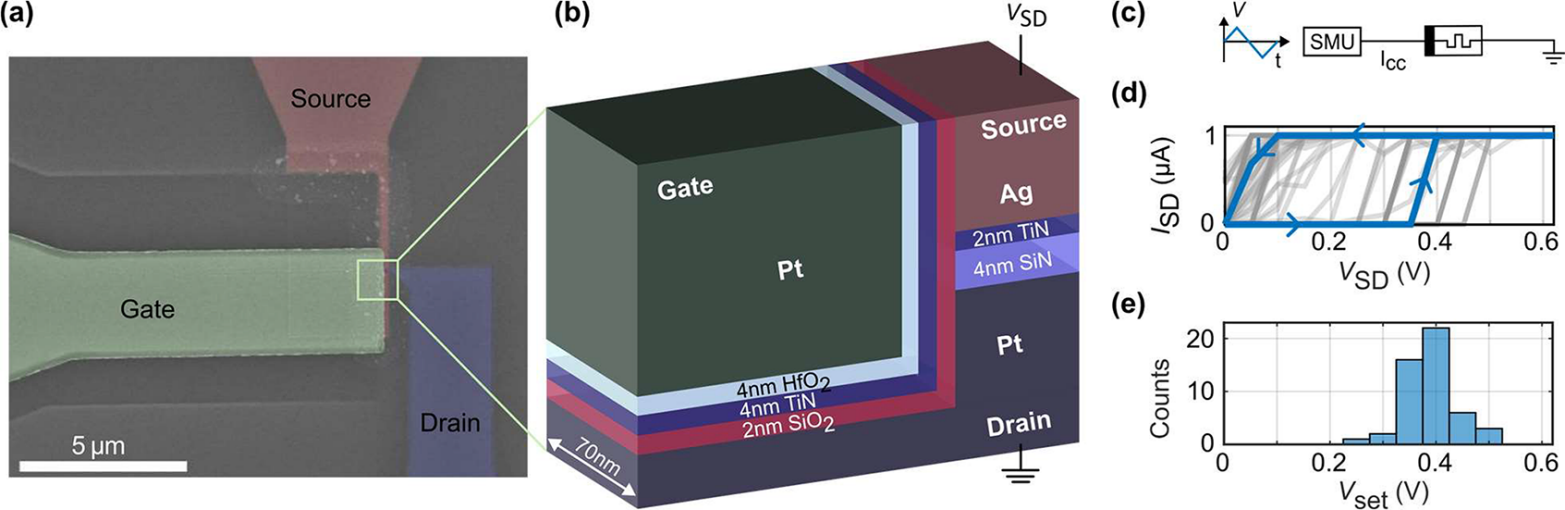
Three-terminal (3T) memristor overview and 2T operation. (a) False-color top view scanning electron microscope image of the device. The “gate” electrode (Pt) is on the left, in green; the active “source” electrode (Ag) comes from the top, in red; and the “drain” electrode (Pt) is on the right, in blue. (b) Schematic device cross section. Between the Ag source and Pt drain, a bilayer nitride layer (2 nm TiN on 4 nm SiN) is used to confine the ion migration in the 2 nm SiO_2_ layer toward the Pt gate. A 4 nm TiN, 4 nm HfO_2_ bilayer is used to prevent ion migration toward the gate. The bottom Pt drain has a width of 70 nm confining the device-under-testing (DUT). (c) Schematic of the measurement setup and input signal. A triangular voltage sweep is applied to the source with a source-measure unit (SMU) that limits the current to a compliance current (*I*_cc_) and measures the current through the memristor. (d) DC IV characteristics of 50 overlaid cycles without the gate (floating), focused on the region of interest. In blue, the median value of all measurements is shown. (e) Histogram of extracted *V*_set_ at 90% *I*_cc_.

Our tunable 3T memristor with nanoscale dimensions (0.07 μm^2^ footprint) is depicted in Figure 1. We first show the device in a 2T operation (IV cycles, ungated) as proof of the memristive origin of the switching mechanism. This plot will further serve as a reference for the gated experiments below.

The presented device is based on a 2T memristor with Ag as the active top electrode, also called the source in this work, and Pt as the inert bottom electrode, the drain, with SiO_2_ as the switching medium. The 3T functionality is enabled by introducing another Pt electrode as a side gate. This additional electrode will allow us to influence the memristor operation by either shifting the set voltage or triggering the resistance change itself between source and drain.

A false-colored top view scanning electron microscope (SEM) image, along with a zoomed-in 3D sketch, is provided in Figure 1(a) and (b) to illustrate the structure of the device-under-testing (DUT). From the cross section, we see that the source and drain are separated by 2 nm TiN and 4 nm SiN. These layers confine the ion migration to the SiO_2_ active region. As can also be seen in Figure 1(b), the device has a width of only 70 nm. Thus, our active region is confined to the nanoscale with a height of 6 nm, width of 10 nm toward the gate, and width of 70 nm from the bottom Pt drain electrode, leading to critical dimensions of 70 nm × 10 nm × 6 nm = 4200 nm^3^. Additional SEM images can be found in the Supporting Information, Figures S1 and S2.

The in-house fabrication starts with a standard Si wafer with 200 nm of thermally grown SiO_2_ on top. The drain is patterned by electron beam lithography (EBL). Subsequently, we etch 50 nm into the SiO_2_ with reactive ion etching (RIE) and deposit 3 nm Ti and 47 nm Pt using electron-beam evaporation (EBE). Afterward, a nitride bilayer (4 nm SiN, 2 nm TiN) is grown with atomic-layer deposition (ALD) to avoid filament formation directly between the drain and source. Next, the source is patterned by EBL and deposited using EBE (1 nm Cr, 24 nm Ag, 17 nm Pt, 3 nm Cr). To allow for filament formation at an interface, the gate region is patterned with the EBL and subsequently physically etched by ion bombardment. Next, the switching medium (2 nm SiO_2_) as well as a blocking layer of 4 nm insulating TiN and 4 nm HfO_2_ are deposited via ALD. The gate is patterned with EBL using an MMA/PMMA double-layer resist and deposited with EBE at an angle. Finally, after a last photolithography step, all buried electrodes are opened by removing the covering dielectrics using RIE.

To prove the memristive behavior of the fabricated devices, we first characterize their 2T (source–drain) part. DC measurements are carried out by grounding the drain, applying a voltage *V*_SD_ on the source, and letting the gate float. In Figure 1(b), the contacted electrodes and in Figure 1(c) the setup are schematically shown. After initial formation with a floating gate and a compliance current *I*_cc_ = 10 nA, we sweep *V*_SD_ from 0 V to 4 V to −2 V and back to 0 V with a sweep rate of 0.05 V/step and repeated it in 50 cycles. We used this extended voltage range to capture all of the switching events. In Figure 1(d), the regions of interest containing the set voltage of all 50 measurements are overlaid on top of each other with the median value shown in blue. The full sweep data can be found in Supporting Information Figure S5(a). The set voltage *V*_set_, at which the devices turn on, is defined as the point where the current reaches 90% of the compliance current *I*_cc_ = 1 μA. We plot the distribution of *V*_set_ as a histogram in Figure 1(e). A narrow distribution can be observed with an average *V*_set_ well below 1 V (mean of 0.39 V, median of 0.40 V, and standard deviation of 0.05 V). We find the ON-resistance to be 1.65 MΩ, which is in line with the literature.^13,42,43^ Additional pulsed set time and retention measurements show a fast 2T set of around 1 μs and a volatile operation. These measurements can be found in the Supporting Information, Figures S3 and S4, respectively.

### Gate-Induced Operation Point Tuning

To explore the potential of the 3T memristor, we begin by applying a constant gate voltage offset while still performing DC IV measurements between the source and drain electrodes. This allows us to gain insight into the possibility of adapting the operation point with the gate contact and thereby increasing the range of applications of the device.

**Figure 2:**
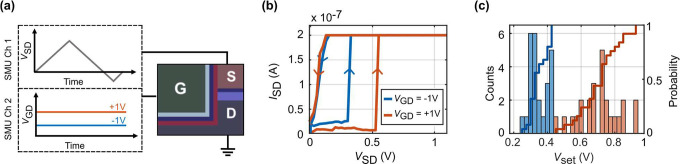
“Set-voltage-tuning” mode. (a) Measurement schematic. A triangular voltage signal is applied to the source (*V*_SD_ in dark gray), while the drain is grounded. During consecutive cycles, the gate voltage is alternated between two offsets, *V*_GD,1_ = −1 V (blue) and *V*_GD,2_ = +1 V (orange). (b) DC IV characteristics showing a representative cycle of each *V*_GD_ offset, i.e., one corresponding to the median value of *V*_set_ from the 50 measurements shown in (c), focused on the region of interest. The difference between *V*_set,1_ = 0.325 V (blue) and *V*_set,2_ = 0.55 V (orange) corresponds to a 69.2% shift in *V*_set_. (c) Histogram of 50 alternating measurements for *V*_GD,1_ = −1 V (blue) and *V*_GD,2_ = +1 V (orange). *V*_set_ is evaluated at the point where the current reached 90% of the compliance current *I*_cc_ = 200 nA.

To demonstrate the gate-induced operation point tuning, we apply the voltage signals, as shown in Figure 2(a). The essential difference from the 2T operation is that the gate electrode is now alternated between two constant offsets. On the Ag top electrode (source), we apply a triangular-shaped voltage sweep from 0 V to 1.1 V and back, with a sweep rate of 0.025 V/step using a source-measure unit (SMU, Keysight B2912a) and a current compliance of *I*_cc_ = 200 nA. The Pt bottom electrode (drain) is grounded. On the Pt gate electrode, two different voltages, *V*_GD,1_ = −1 V and *V*_GD,2_ = +1 V, are applied, represented by the blue and orange curves in the *V*_GD_ plot, respectively. To account for potential shifts of the memristor over successive cycles, we alternated *V*_GD_ between consecutive cycles. In total, we carried out 50 *I*–*V* measurements, which corresponds to 25 full cycles per gate offset. Two representative measurement cycles are listed in Figure 2(b). The full measurement data can be found in Supporting Information Figure S5(b). By applying a gate voltage *V*_GD,1_ = −1 V (shown in blue), the set voltage is lower than in the ungated experiments previously discussed and has a value of *V*_set,1_ = 0.325 V. Conversely, when we apply a gate voltage *V*_GD,2_ = +1 V (shown in orange), the set voltage shifts to a value of *V*_set,2_ = 0.55 V. This corresponds to a shift in the operation point of 69.2% due to the gating of the device. The measurements shown in Figure 2(b) are characterized by *V*_set_ that corresponds to the median of the 25 full cycles per gate voltage. To complete the analysis, we extracted the set voltages of all cycles at 90% *I*_cc_ = 200 nA and plotted this quantity in a histogram in Figure 2(c). The gate voltage creates two well-distinguishable states without any overlap between their distributions. The cumulative probabilities of the distributions are plotted in the same figure. We evaluate the ON-resistance in both cases as well: For *V*_GD,1_ = −1 V, we find 640 kΩ, and for *V*_GD,2_ = +1 V, we find 1.28 MΩ. Additionally, the reset voltage analysis as well as continuous gate sweep measurements can be found in the Supporting Information, Figures S6 and S7, respectively.

**Figure 3:**
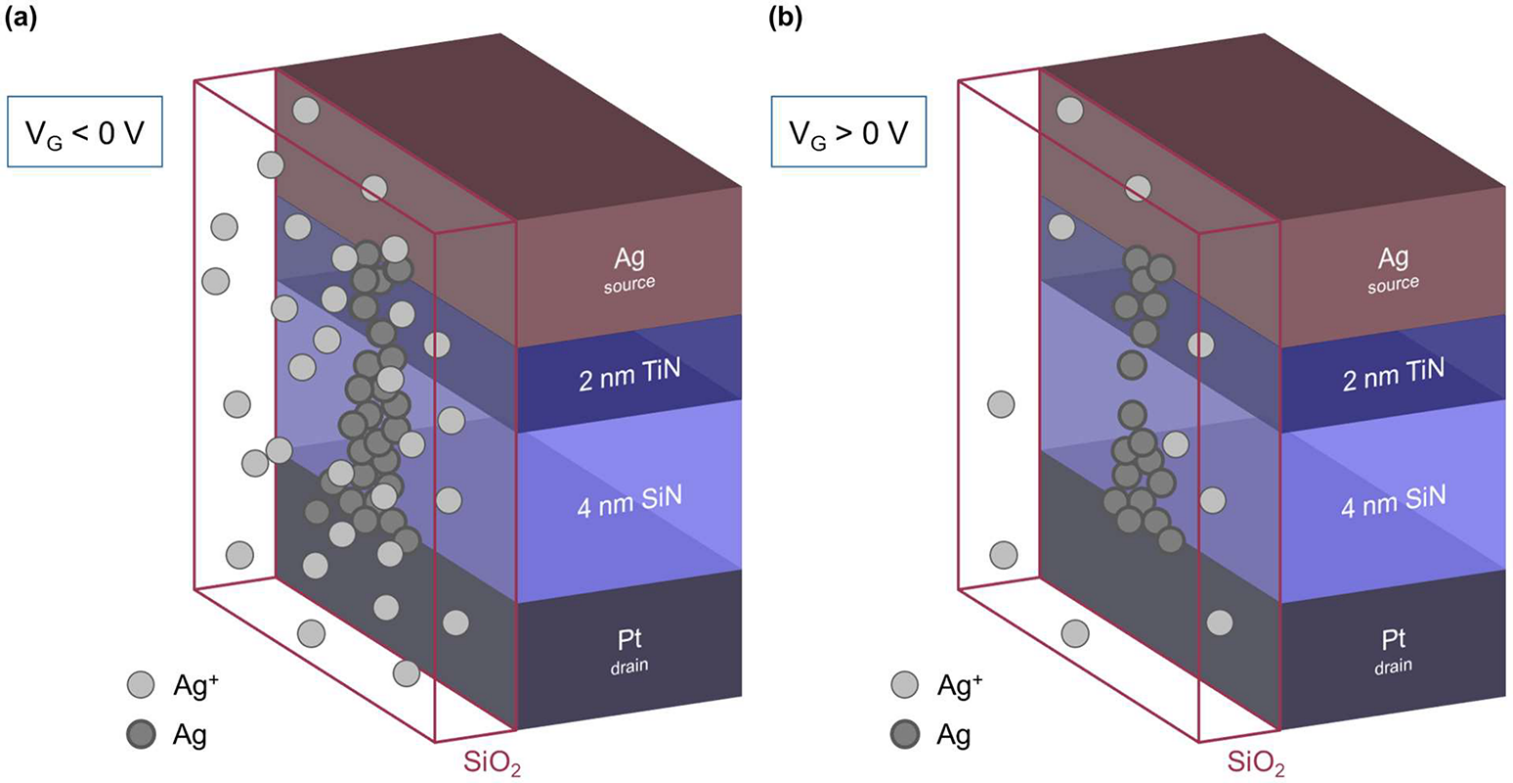
Visual illustration of the operation mechanism. Inside the outlined red box, representing the SiO_2_ layer, Ag atoms and ions are depicted in two shades of gray. (a) Applying a negative gate voltage is assumed to increase the silver ion concentration and facilitate filament formation. (b) Applying a positive gate voltage is assumed to decrease silver ion concentration and hinder filament formation.

Adding the gate voltage enables us to shift the set voltage up and down compared to the ungated case. This hints at the gate voltage playing a role in facilitating or hindering filament formation in the memristor depending on the polarity of the applied voltage. A gate voltage influences the electric field within the SiO_2_ and at the interfaces of the remaining two electrodes. We assume that this changes several reaction steps necessary for filament formation: oxidation at the Ag electrode, distribution of the silver ions inside SiO_2_, and reduction of silver ions. This in turn will influence the filament formation dynamics. This interplay between gate voltage and filament reactions allows us to tune the set voltage or initiate the resistance change, as discussed below. We assume that the negative gate voltage increases the rate of Ag^+^ ion formation, thereby increasing the silver reduction rate at the filament, which corresponds to a lower *V*_set_. In contrast, the positive gate voltage hinders the ion formation, thereby reducing the reduction rate at the filament and causing an increase. These effects are visualized in Figure 3.

### Gate-Triggered Resistance Change

In the following, we show that the resistive state of our 3T memristor can be changed by modulating the gate bias. For this purpose, the input voltage on the source and a series resistor is fixed, and the gate voltage is varied. Furthermore, the measurement method is changed from DC IV cycles to 1 kHz pulsed operation to show the dynamical response of the device and allow for comprehensive endurance measurements.

**Figure 4:**
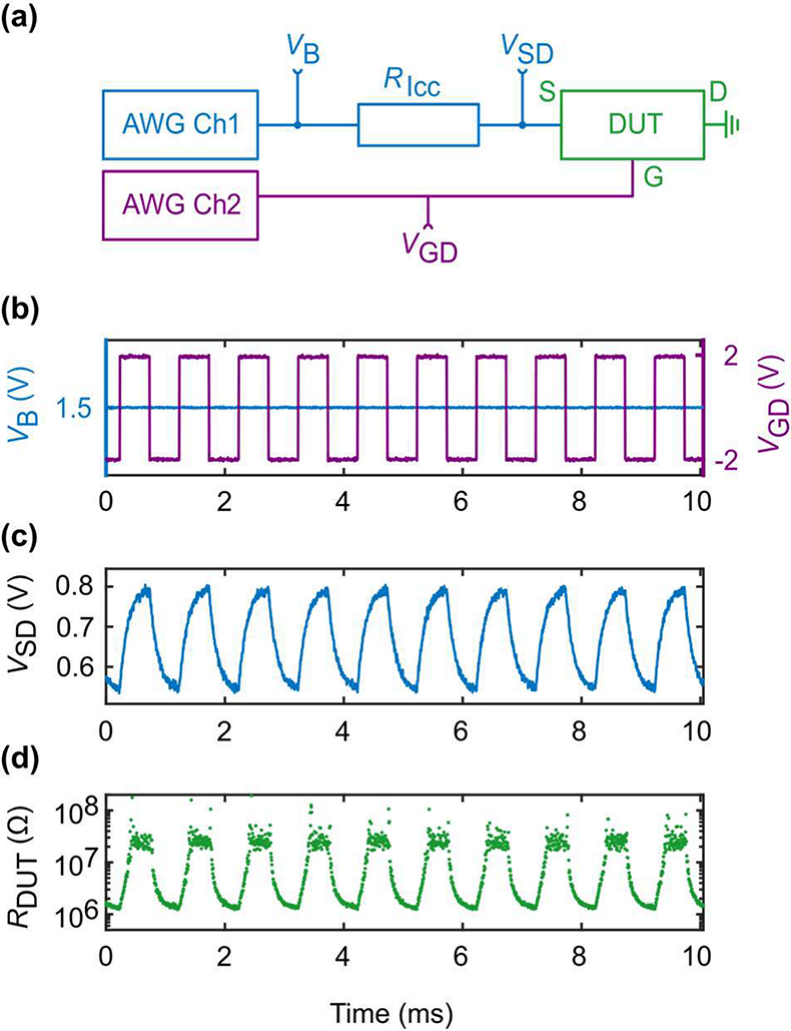
Representative 1 kHz memristor state modulation by applying gate voltage pulses. (a) Measurement schematic showing the bias path in blue, the device-under-testing (DUT) in green, and the gating path in purple. An arbitrary waveform generator (AWG) supplies the constant bias voltage *V*_B_ and the pulsed gate voltage *V*_GD_. The DUT consists of three contacts: source (S), gate (G), and the drain (D, ground). The bias path consists of AWG channel 1 and a current-limiting resistor (*R*_Icc_) that connects to the source of the DUT. The gate path consists of AWG channel 2, which is connected to the gate of the DUT. (b) Input voltages from the AWG measured at *V*_B_ (blue) and *V*_GD_ (purple). The input bias voltage *V*_B_ is held constant at 1.5 V, while the gate voltage is modulated between *V*_GD_ = ±V with a 1 kHz frequency. (c) Measured source–drain voltage *V*_SD_. We observe a typical response for capacitive charging overlaid onto the resistance change of our memristor. (d) Device resistance (*R*_DUT_) converted from *V*_SD_. *R*_DUT_ follows the shape of *V*_GD_; the higher the *V*_GD_, the higher the device resistance, and vice versa.

The experimental setup is schematically shown in Figure 4(a). Two input signals are supplied by an arbitrary waveform generator (AWG, Agilent 33500B) and monitored with an oscilloscope (Keysight MSO9104A), which is also used to evaluate the state of the device-under-testing (DUT).

The AWG channel 1 supplies a constant bias voltage *V*_B_, which is monitored with an oscilloscope. Next, a resistor *R*_Icc_ = 1 MΩ is inserted between AWG channel 1 and the DUT to limit the current and protect the DUT. Between *R*_Icc_ and the source contact, we read out the source–drain voltage *V*_SD_ across the DUT. The drain contact is grounded. The bias path is shown in blue in Figure 4(a), and the DUT in green. The resistance between source and drain together with *R*_Icc_ acts as a voltage divider, enabling us to calculate the resistance *R*_DUT_ of the DUT. Channel 2 of the AWG supplies the square-shaped gate voltage pulses with 1 kHz frequency, displayed in purple in Figure 4(a). It is read out with the oscilloscope as *V*_GD_.

A representative measurement is shown in Figure 4(b)–(d). The input bias voltage is held constant at *V*_B_ = 1.5 V, while the gate voltage is modulated between ±2 V, as depicted in Figure 4(b) in blue and purple, respectively. The gate voltage is increased compared to that before to account for the faster measurement speed. The voltage between source and drain, *V*_SD_ (Figure 4(c)), exhibits a capacitive charging response, which results from the high resistances present in the circuit, thus bringing the setup close to its RC limit. The increased voltage *V*_SD_ compared to the set voltages of the *I*–*V* measurements stems from the pulsed operation happening on a faster time scale and is commonly known as the voltage–time dilemma.^44^ The device resistance, *R*_DUT_, is extracted as discussed before from *V*_SD_ and is reported in Figure 4(d) in green. The resistance of the device follows the shape of the gate voltage: a higher gate voltage increases the DUT resistance, whereas a lower gate voltage decreases it. This indicates that the gate voltage may influence the filament growth between source and drain, a negative gate voltage favoring this process, while a positive one hindering it. By evaluating the data at the last point prior to the gate voltage transition, we extract an average high-to-low resistance ratio of 18.9. The retention of the gated resistance states is shown in Figure S8.

Leakage current measurements to the gate with the same voltages as used in this work (±1 and ±2 V) were carried out. Leakage currents were found to be ≤1.6 nA, hence significantly smaller than the source–drain current in compliance (*I*_SG_, *I*_DG_ ≪ 100 nA ≤ *I*_SD,cc_). The full analysis can be found in Table S2.

We prioritized reliability and endurance during the aforementioned measurements and therefore chose high resistance values in this experiment, which leads to a high RC time constant and, accordingly, to a slow response (see Methods section “Pulsed Measurements”). To ensure a precise data evaluation, we determine the resistance state at the end of each pulse. Further investigations using lower resistances and therefore allowing for higher speeds are of high interest, too. They would require the DUT to withstand higher currents without degradation, which has yet to be explored.

### Endurance and Reproducibility

Bringing the 3T device into applications requires long device endurance, reproducible operation, and low device-to-device variability.

**Figure 5:**
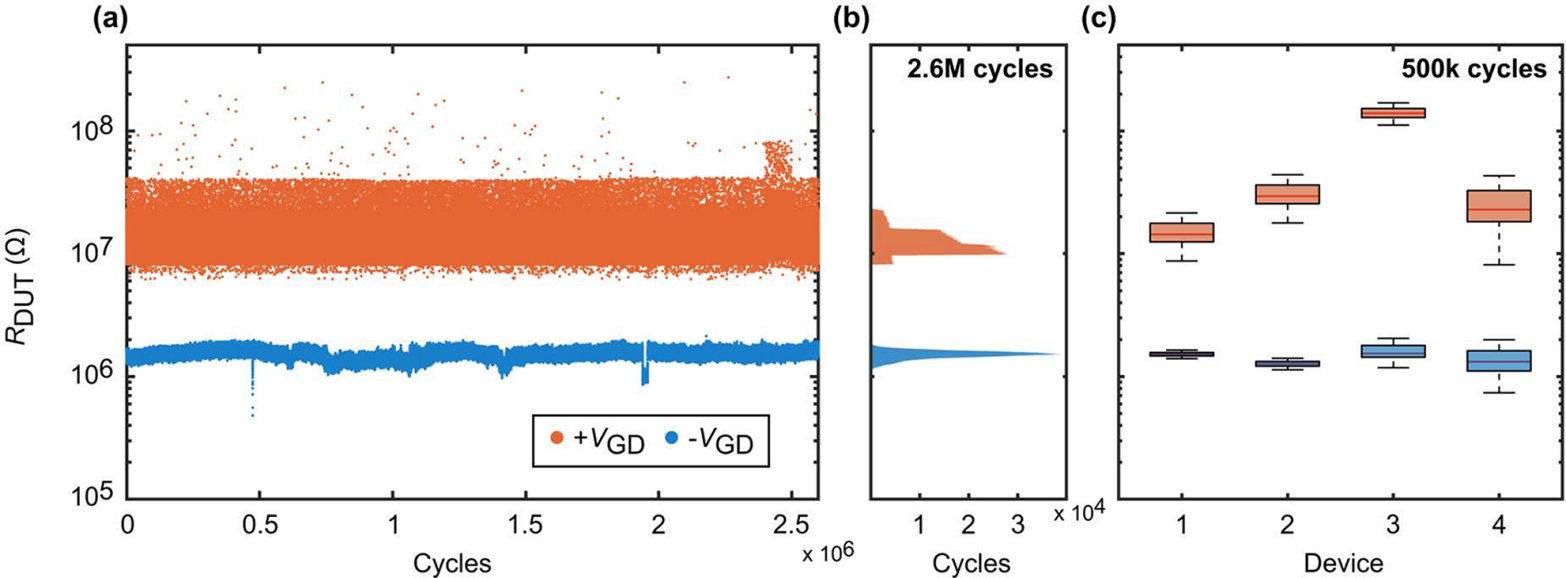
Endurance and device-to-device variability. (a) *R*_DUT_ plotted over 2.6M cycles. Each cycle consists of a positive and a negative 500 μs voltage pulse applied to the gate with *V*_GD_ = ±2 V. *R*_DUT_ is evaluated at the last point of the pulse. The experiment is performed, and the data are collected in packs of 100k pulses. (b) Associated histogram shows a mean of 1.5 MΩ (median of 1.5 MΩ) for the negative gate pulse (blue) and a mean of 12.7 MΩ (median of 12.0 MΩ) for the positive gate pulse (orange), respectively. (c) Comparison of *R*_DUT_ measured over 500k cycles for four devices displaying minor device-to-device variation. Results from device 1 were used in all previous figures.

We first conducted endurance measurements by collecting a total of 2.6 million cycles in packs of 100 000 cycles, using the same device as in all previously shown measurements and labeled “Device 1”. In between data recordings, the device is continuously cycled. As a result, the actual number of cycles is even larger than that shown here. We read out the device state immediately before the gate voltage *V*_GD_ transition from low to high or high to low. Hence, each cycle contains a data point for the positive and negative pulse. The same setup as that in Figure 3(a) is used. Figure 5 displays the resistance of the device under test (*R*_DUT_) over the 2.6 million cycles in subplot (a). To further analyze the data, the corresponding histogram is shown in Figure 5(b). This representation clearly indicates that there is no overlap between the two gated states. We find that for a gate voltage of *V*_GD_ = −2 V, the device resistance has a mean of 1.5 MΩ (median of 1.5 MΩ) with a standard deviation of 0.12 MΩ. In the case of *V*_GD_ = +2 V, we extract a mean of 12.8 MΩ (median of 12.0 MΩ) and a standard deviation of 3.2 MΩ. Using the mean, we found a ratio of 8.5. The results show that the device reliably switches over the entire 2.6 million cycles. We did not encounter a single failure, demonstrating its excellent endurance capabilities.

Second, we evaluated the performance of multiple devices to investigate the reproducibility of the 3T memristor. For this purpose, we again use Device 1 and compare it with three other devices. We gather 500 000 data points per device to ensure large enough statistics for a fair comparison of all devices. Different voltage settings are used for each device so that the low resistance states are well aligned (see Table 2 in the Methods section “Pulsed Measurements”). The data collection is done in the same way as before, and the results are plotted in Figure 5(c). Each device exhibits two distinct states with a narrow distribution around their mean value. Additionally, the variability between devices is low, which allows us to define a common threshold to distinguish the two states for all devices. This demonstrates the repeatability and consistency of the device’s gated operation concept.

### Application: Pseudorandom Bit Sequences

To evaluate further the device’s performance under practical application scenarios, we conducted tests using pseudorandom bit sequence (PRBS) data. A PRBS of order *k* is a sequence of pseudorandom bits of length of 2^*k*^ – 1. It contains shorter sequences of equal bits of various lengths. This mimics the possible bit combinations that might occur in a practical scenario, similar to the MNIST data set. In our experiments, the bits were translated to *V*_GD_ = ±2 V. The PRBS test serves as a benchmark to measure the device’s ability to handle and withstand dynamic inputs as used in high-speed data transmission or computation applications. Furthermore, it allows investigating the device’s resilience to random data and potentially long sequences of the same input without deteriorating in quality.

**Figure 6:**
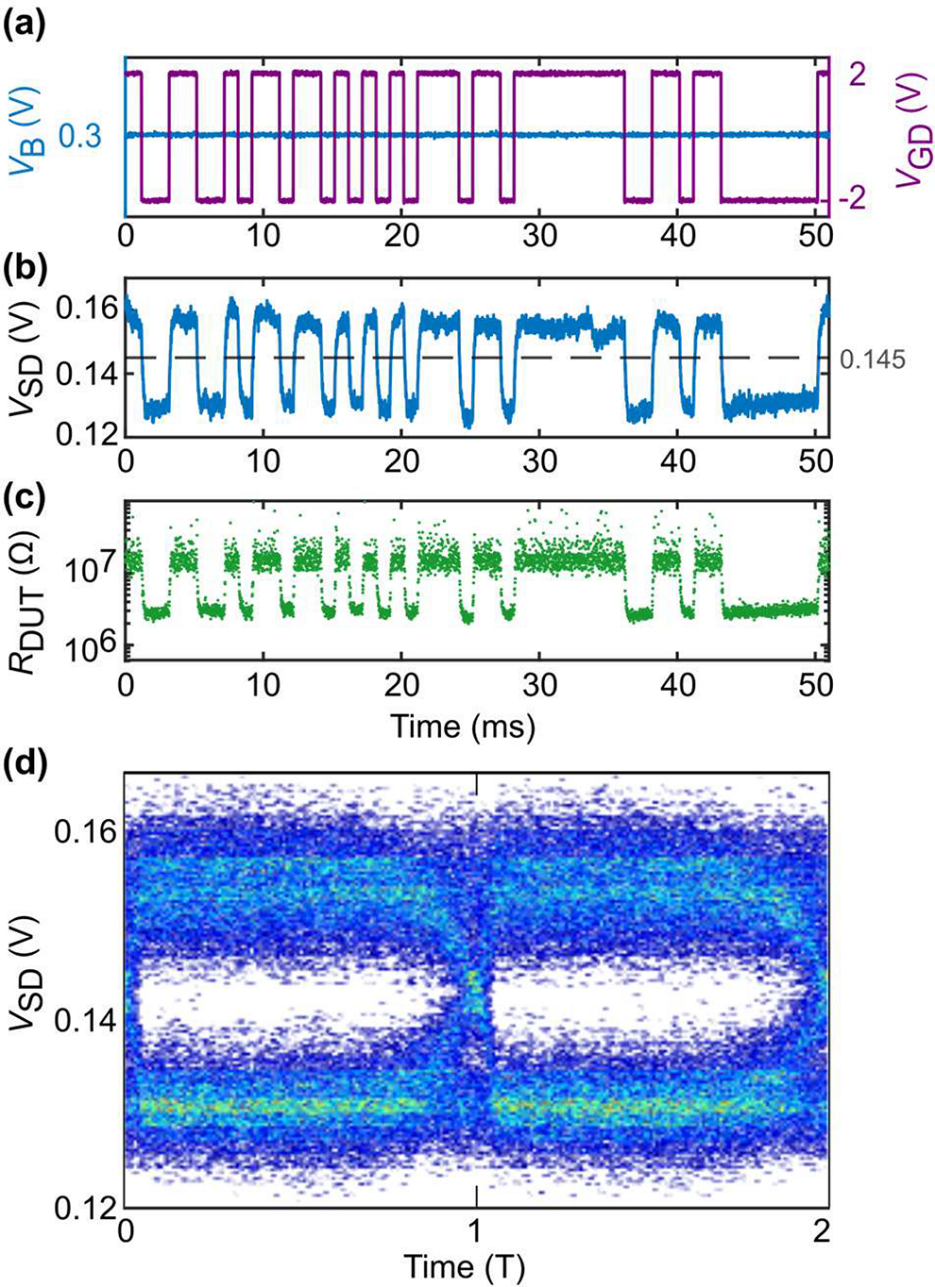
Pseudorandom bit sequence measurements: Representative data and eye diagram of a full data set. (a) Using the same setup as in Figure 3(a), we apply *V*_B_ = 0.3 V (blue) and *V*_GD_ = ±2 V (purple). (b) Measured source–drain voltage, *V*_SD_. The threshold voltage *V*_th_ = 0.145 V is marked with a gray dashed line. (c) Corresponding device resistance *R*_DUT_. (d) Eye diagram based on 202 000 cycles using *V*_SD_ to show the switching dynamics over two periods (T) of measurement data.

The gate voltage (*V*_GD_) is coded as +1 for positive values and −1 for negative values. An exemplary input signal is illustrated in Figure 6(a). Each single data point is encoded by a 1 ms pulse. Similarly, the DUT state is coded depending on the measured *V*_SD_ compared with a threshold voltage. We code the output as +1 for *V*_SD_ > *V*_th_ and −1 for *V*_SD_ < *V*_th_. From the measurement in Figure 6(b), it can be deduced that *V*_th_ = 0.145 is a suitable choice. By comparing the two data strings (input encoded in *V*_GD_ and output read out from *V*_SD_), we calculated the success rate. For the example shown in Figure 6, it is 100%. The resistance of the DUT in this example is plotted in Figure 6(c) for completeness and comparison.

For a complete picture with longer data sets, we repeat these measurements in packs of 50 000 bits for a total of 250 000 bits and evaluate the DUT state again at the last point before the *V*_GD_ flank. We repeated this measurement for four PRBSs of different order (PRBS-7, PRBS-9, PRBS-11, and PRBS-13). By comparing the PRBS input data with the extracted device state, we can calculate the success rate. It varies between 96.6% and 97.4% depending on the PRBS used, as can be seen in Table 1. We observe no degradation in performance for higher PRBS orders and a stable threshold voltage (*V*_th_).

**Table 1:** Pseudorandom Bit Sequence Measurements^a^

	PRBS			
	7	9	11	13
Success rate	96.6%	97.3%	97.4%	97.1%
*V*_th_	0.145	0.145	0.145	0.145

a Success rate and threshold voltage for PRBS of orders 7, 9, 11, and 13.

**Table 2:** Bias Voltage*V*_B_ and Gate Voltage*V*_GD_ Applied to the Four Devices Shown in Figure 4(c)

	Device			
	1	2	3	4
*V*_B_ (V)	1.8	1.5	2	2
*V*_GD_ (V)	±2	±2	±3	±1.5

The eye diagram (Figure 6(d)) is a visual representation of the signal quality and shows the transitions between and fluctuations within the two states. To obtain such an eye diagram, all periods of the measurement data are overlaid. In our case, the period is *T* = 1 ms. The eye is found to be wide open for a PRBS-13 input of 202k cycles. We found an eye height^45^ of 6 mV and an eye opening of 6.3 σ. This implies a high level of noise immunity. The switching times are found to be well within the cycle period, indicating that the device has the potential to operate at higher speeds than the current 1 kbit/s.

## Conclusion

Our work successfully demonstrates the development and characterization of a three-terminal memristor with an ultrasmall footprint of 0.07 μm^2^ and critical dimensions of 70 nm × 10 nm × 6 nm. We show how a gate voltage applied to the device can be used to manipulate the operation point, as evidenced by DC characterization, or initiating the resistance switching, as demonstrated by 1 kHz pulsed measurements. DC characterization shed light on the underlying memristive operation of the proposed device concept with a median set voltage of 0.4 V (ungated case). When a gate voltage is applied, we observe a set voltage shift from 0.325 V (−1 V gated) to 0.55 V (+1 V gated), corresponding to a shift of 69.2%. Pulse measurements of 1 kHz are carried out with the gate as the driving force to change the device resistance. We reported an endurance of over 2.6 M pulses with two distinct resistance distributions. Using the same type of measurement, we compare four devices for 500k pulses and find only minor device-to-device variations. To demonstrate the device’s potential in practical applications, under random inputs, we test its response to PRBSs of varying orders from 7 to 13. Our measurements indicate reliable operation with an open eye diagram and success rates of approximately 97%.

These promising results enable a range of potential applications. In the realm of neuromorphic computing, our device should facilitate spike probability tuning in artificial neurons and the adjustment of synaptic weights. Furthermore, in addition to 2T-memristive devices,^11^ 3T-memristive devices are attracting attention for applications to reservoir computing.^46^ Here, the nonlinear electrical response of 3T-ion-gating transistors plays a crucial role in mapping inputs to a high-dimensional feature space. The time scales and voltages utilized in this work also suggest potential for Internet-of-Things applications. Finally, our findings are promising candidates for the development of memristive logic systems.

## Methods

### Fabrication

First, the bottom electrodes (drain) are patterned using PMMA on a 2 × 2 cm^2^ chip. Then, using RIE, 50 nm deep trenches are etched and subsequently filled with EBE of Pt at 10^–6^ mbar. After lift-off, the chips are polished. Two nitride layers are deposited using ALD: first 4 nm SiN at 300 °C and then 2 nm of TiN at 400 °C. Next, the top electrode (source) is patterned using a resist bilayer of MMA/PMMA. This top electrode consists of 1 nm Cr, 24 nm Ag, 17 nm Pt, and 3 nm Cr deposited by EBE. To remove material for the gate electrode, the chip is coated with a negative resist and patterned. After developing, etching at 20° allows the gate to be contacted from the side. Three ALD layers are deposited next: 2 nm SiO_2_, 4 nm TiN, and 4 nm HfO_2_. The SiO_2_ is the switching medium, and the other two are there to isolate the gate from the two other electrodes. To assess the functionality of the different layers used, we fabricated test chips, stopping the fabrication after the top electrode deposition. Consequently, two-terminal devices using Ag/SiN/TiN/Pt without any physically etched interface were produced. The forming voltage of these test devices was measured and found to be *V*_f_^2T^ = 4.1 V. Evaluating the forming voltage of all measured three-terminal devices, we find *V*_f_^3T^ = 2.7 V < *V*_f_^2T^. This reduction in the forming voltage strongly suggests that the switching phenomenon occurs at the interface, specifically within the SiO_2_ layer. The gate electrode is then patterned using another bilayer resist and by depositing 10 nm Ti and 90 nm Pt under an angle at 10^–6^ mbar with EBE. Finally, the electrode pads are made electrically accessible with an etch step.

### DC IV Measurements

The DC IV measurements are carried out using an SMU from Keysight (B2912a). It is controlled via MATLAB, where the compliance current, the step size, the range, the integration time, and other parameters can be set. We applied a compliance current to protect the DUT. Alternatively, a series resistance could be used. The device is connected with two 50 μm pitch probes, one GSG connected to drain–source–ground pads and a GS probe connected to the same ground pad and the gate to ensure the same ground level on both input signals. In the forming stage and for the initial electrical characterization of the source–drain contact, the second probe is left disconnected. The triangular voltage sweep on source–drain electrodes goes from 0 to 4 V, down to −2 V, and back to 0 V in steps of 0.05 V with a compliance current of 1 mA and integration time of 60 ms for 50 cycles. To test the gate influence, we repeated 50 measurements, this time with the second probe connected. The range of source–drain was set to [−2 V, 2 V] with a step size of 0.025 V, integration time of 40 ms, compliance current of 200 nA, and alternating the gate voltage from −1 V to +1 V between every cycle. This was repeated 25 times per gate voltage. The set voltage was extracted at the first measurement point after the current reaches 90% of the compliance current. The difference in the compliance currents is not significant for the evaluation, as the extracted values, such as the set voltage, are not directly compared to each other.

### Pulsed Measurements

Subsequently, we describe the measurement setup for determining the values plotted in Figures 4 and 5. We also comment on the setup’s RC time and how to derive the memristive resistance *R*_DUT_.

**Figure 7:**
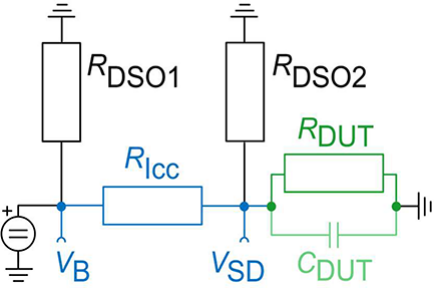
Circuit representation of the setup. *R*_DSO1_ and *R*_DSO2_ are the input impedances of the digital storage oscilloscope (DSO) channels.

The setup given in Figure 4(a) has as a voltage source an arbitrary waveform generator (Agilent 33500B) that supplies two signals (*V*_B_ and *V*_GD_). *V*_B_ is a constant bias voltage, while *V*_GD_ is a rectangular pulse signal applied to the gate with a 1 kHz frequency. We monitor the voltages in the setup with an oscilloscope (Keysight MSO9104A). The constant bias voltage *V*_B_ is read out by the oscilloscope on channel 1 with an input impedance *R*_DSO1_ = 1 MΩ. The oscilloscope channel 2, with an input impedance *R*_DSO2_ = 1 MΩ, monitors the voltage over the device (*V*_SD_). Finally, channel 3 of the oscilloscope reads the rectangular voltage (*V*_G__D_) supplied to the gate. To limit the currents and protect the device, a series resistance *R*_Icc_ = 1 MΩ is added. A simplified setup is given in Figure 3(a). However, for simplicity, we omitted the three internal oscilloscope impedances in said figure. An equivalent circuit representation of the source–drain path across the device is shown in Figure 7. In this figure we also show the input impedances of the oscilloscope, which can be internally set to 1 MΩ

To calculate the limit of our pulsed measurement setup, we evaluate the RC limit. The capacitance per meter of the cables in the setup is *C*_BNC_ = 80 pF/m. Together with the used series resistance of *R*_Icc_ = 1 MΩ and the two meters of cable, we arrive at an RC time constant of τ_RC_ = 160 μs for the setup. This time constant is observed in the slope of the voltage response *V*_SD_ of the pulsed measurements shown in Figure 4(c) and Figure 6(b). Thus, the setup limits further investigation of the potential speed of our devices.

Subsequently, we show that the potential influence of the device capacitance *C*_DUT_ is low. We calculate the device impedance . The capacitance can be estimated using , and the permittivity *ε*_r_ = *ε*_r__,SiN_ = 7.5, ε_0_ = 8.85 × 10^–12^ F m^–1^, the area *A* = 70 nm × 1.5 μm, and the distance *d* = 6 nm. We find *C*_DUT_ = 1.16 × 10^–15^ F. Considering the small value of *C*_DUT_ and ω = 1 kHz, the influence of the capacitance is 12 orders of magnitude smaller than the resistive term. Hence, *Z*_DUT_ ≈ *R*_DUT_.

Therefore, we can derive the device resistance from Figure 7 and omit *C*_DUT_.
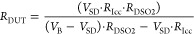


Due to the high resistances in the system, the noise limit of the setup is reached and unphysical values can be found due to the low currents flowing into the oscilloscope. To be able to subtract the potential noise fluctuation, we evaluated the noise level as noise = 2.4·σ(*V*_B_), as *V*_B_ being constant allows for an easy fit. To account for data points below this limit, we shifted these points accordingly.

However, the thermal noise in the measurement setup can add up and may lead to the wrong interpretation of *R*_DUT_ given by the formula above. Thermal noise, e.g., across *V*_SD_, may render the expression for *R*_DUT_ infinitely large or negative, which clearly would not be physically correct. This is because the denominator in the expression above simplifies to (*V*_B_ – 2*V*_SD_)*R*_Icc_ as *R*_Icc_ = *R*_DSO2_ = 1 MΩ. This expression becomes negative if *V*_SD_ ≥ *V*_B_/2. This happens upon a small flow of *V*_SD_ as *V*_SD_ approaches *V*_SD_ = *V*_B_/2. To avoid such overestimated or nonphysical resistance values, we subtract the noise term from the measured *V*_SD_. The noise in the setup can be found by performing a noise measurement at *V*_B_. The noise measurement across *V*_B_ is easily obtained, as the voltage applied across *V*_B_ is constant. The noise found at *V*_B_ is identical to the one at *V*_SD_ if *R*_DUT_ is large. In this case, the equivalent circuit in Figure 7 is symmetric around *V*_B_ and *V*_SD_, as *R*_Icc_ = *R*_DSO2_ = 1 MΩ. By experiment, we find around the measurement point *V*_B_ for the noise a standard deviation of σ(*V*_B_). To be on the safe side and cover 99% of the signal distribution, we correct *V*_SD_ by subtracting a noise on the order of 2.4·σ(*V*_B_).

All of the measurements in the paper have been performed with one device, called “Device 1”. Yet, to get device-to-device variation measurements in Figure 5(c), we performed 500k cycle measurements on three more devices. The voltages *V*_B_ and *V*_GD_ were chosen in such a way that the low resistance state would be comparable for all four devices. The exact values for all four devices shown in Figure 5(c) are given in Table 2.

The eye diagram is obtained by overlaying all periods of the measurement data. We calculate the eye height and eye opening by fitting two normal distributions to the data and extracting the respective mean values μ_0_, μ_1_ and standard deviations σ_0_, σ_1_. The eye height is defined as the difference between the means subtracted by 3 standard deviations.
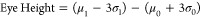


The eye opening is defined as the ratio of the difference in the means and the larger standard deviation:
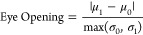


## Data Availability

Data from this work are available upon reasonable request.

## Supporting Information Available

Structural SEM images; 2T set time measurements; 2T retention measurements; full *I*–*V* measurement data; reset voltage analysis; continuous gate voltage measurements; leakage current measurements; gate-influence retention (PDF)

## Accession Codes

Code from this work is available upon reasonable request.
