# Supplementary material for: Versatile Nanoscale Three-Terminal Memristive Switch Enabled by Gating
Source: ACS Nano. 2024 Apr 9;18(16):10798–806. doi: 10.1021/acsnano.3c11373 (PMC11044582; doi:10.1021/acsnano.3c11373)
Supplement: Supplementary file 1 — nn3c11373_si_001.pdf [file nn3c11373_si_001.pdf]

Supporting Information for

Versatile Nanoscale Three-Terminal Memristive Switch Enabled by Gating

Mila Lewerenz,[1] Elias Passerini,[1] Bojun Cheng,[2] Markus Fischer,[1] Alexandros Emboras,[3]*

Mathieu Luisier,[3] Ueli Koch,[1] Juerg Leuthold [1]*

1 ETH Zurich, Institute of Electromagnetic Fields (IEF), 8092 Zürich, Switzerland

2 Microelectronics Thrust, Function Hub, HKUST, Guangzhou, China

3 Integrated Systems Laboratory (IIS), ETHZ, 8092 Zürich, Switzerland

Email Correspondence: mila.lewerenz@ief.ee.ethz.ch, leuthold@ethz.ch

1. Structural Images

Process control chips were fabricated in parallel with the devices presented in the manuscript. They have the same layers and thicknesses as the main chip (from bottom to top: Si / 200 nm SiO_2 / 3 nm Ti / 47 nm Pt / 4 nm SiN / 2 nm TiN / 1 nm Cr / 24 nm Ag / 17 nm Pt / 3 nm Cr). They served to validate the processes such as the physical etching step without the risk of damaging the main chip. A cross section of such a process control chip is shown in Figure S1 and shows the layer stack of 50 nm bottom electrode (BE, Ti/Pt), 6 nm nitrides, 44 nm top electrode (TE, Cr/Ag/Pt/Cr). On top, the remains of the resist, hydrogen silsesquioxane (HSQ), can be seen. The thicknesses were extracted from the SEM image using ImageJ. On the main chip, test structures were fabricated for non-invasive checks during the fabrication process. In Figure S2, such a test structure to measure the width of the bottom electrode and verify the contact pad resistance is shown. The planned width was 70 nm. The measurements were done with the SEM directly and show the actual fabricated devices to be slightly narrower.

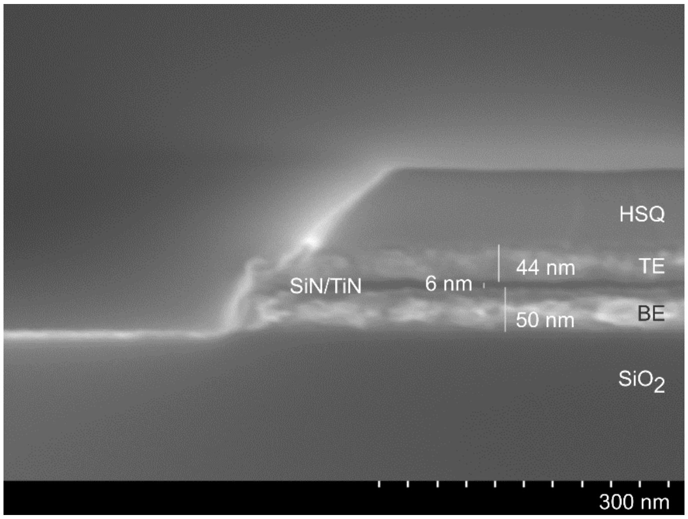

Figure S1: Cross-sectional scanning electron microscopy (SEM) image of a control chip fabricated in parallel with the device chip. It shows the layers (BE/nitrides/TE) with their corresponding thicknesses (50 nm/6 nm/44 nm). A layer of resist (HSQ) is left on-top of the TE. One can see a steep etching of the layers. The thickness extractions were performed with ImageJ.

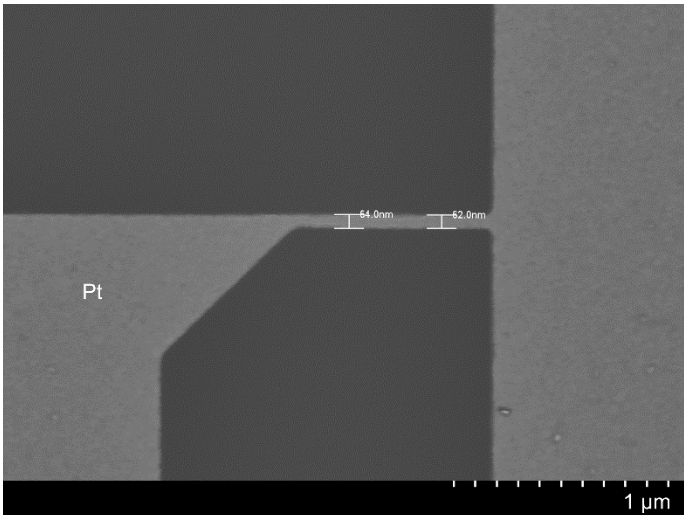

Figure S2: Top view of an SEM image showing the actual measured width of the 70 nm structured bottom Pt wires.

2. Two-Terminal Set Time

We evaluate the set time t_{set} of the two-terminal section of our devices by adding a $10\ \mathrm{k\Omega}$ series resistance and applying input voltage pulses of 4 V. Two read pulses of 0.25 V were used to measure the device state before and after the set pulse. An exemplary measurement is shown in Figure S3. Using the definition laid out by Lübben et al.[1], we evaluate t_{set} as the time between the two rising edges at 50% height. Evaluating t_{set} for 3 devices, we find a mean value of $1.0\ \mathrm{\mu s}$.

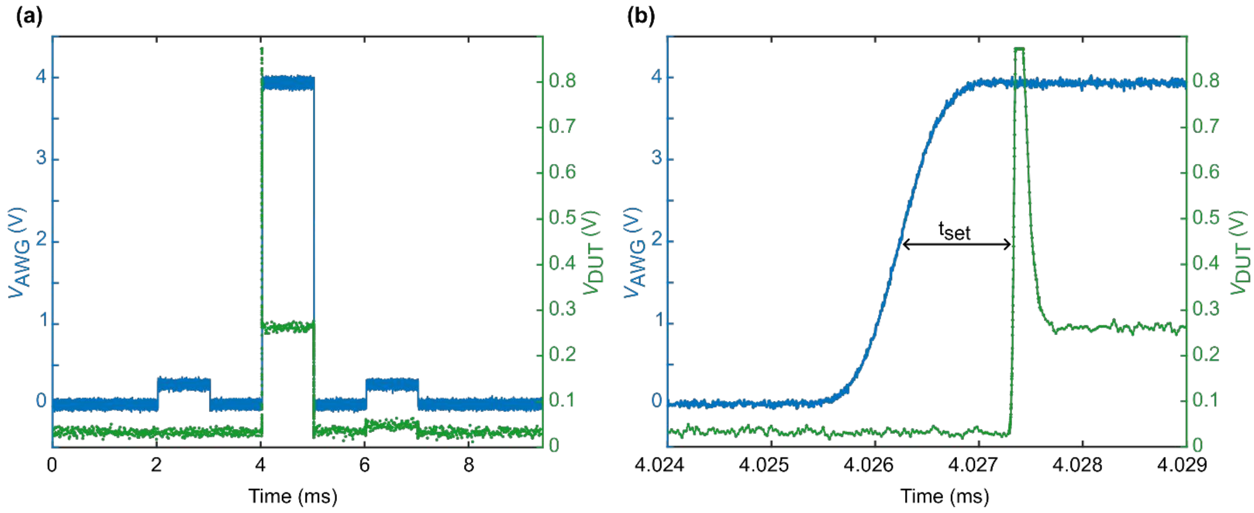

Figure S3: Pulsed set time measurements with a series resistance of 10 kΩ *and an input voltage pulse amplitude of 4* V*. (a) Read-set-read operation. In blue, the incoming voltage pulse is shown. The read pulse has a height of 0.25* V*, the set voltage pulse of 4* V*. In green, the voltage between the series resistance and the device-under-testing is shown. During the first read pulse, the device is in off-state. During the application of the set pulse, the device turns on. Finally, the device is still in the on-state during the second read pulse. Between the flanks, only every 20th data point is plotted for legibility. At the flanks, all data points are shown. (b) A closer look at the set operation. In this example, the set time was evaluated to be* $1.08\ \mu s$.

3. Retention

Pulsed retention measurements were carried out using the same bias voltage $V_{\mathrm{B}} = 1.5\ \mathrm{V}$ and series resistance $R_{\mathrm{S}} = 1\ \mathrm{M\Omega}$ as in the pulsed measurements shown in Figure 4. A transimpedance amplifier (TIA) was used to amplify the current and translate it into a voltage. A read pulse of $0.1\ \mathrm{V}$ amplitude and $1\ \mathrm{ms}$ duration was followed by an intentionally long set pulse of $125\ \mathrm{ms}$ duration to mimic longer operation as used in the manuscript. We measured the state of the device using twenty read pulses ($0.1\ \mathrm{V}$, $1\ \mathrm{ms}$) with 1 ms of wait time in between. We find that the device turns off in less than $40\ \mathrm{ms}$. In Figure S4(a-b), the full retention measurement is shown. In Figure S4(a), the incoming voltage from the arbitrary wave generator (AWG) V_{AWG} is plotted. In Figure S4(b), the voltage after the TIA V_{TIA} is shown. The 20 read pulses are shown in Figure S4(c) with V_{AWG} in blue and V_{TIA} in green. One can see how the device turns off after about $37\ \mathrm{ms}$.

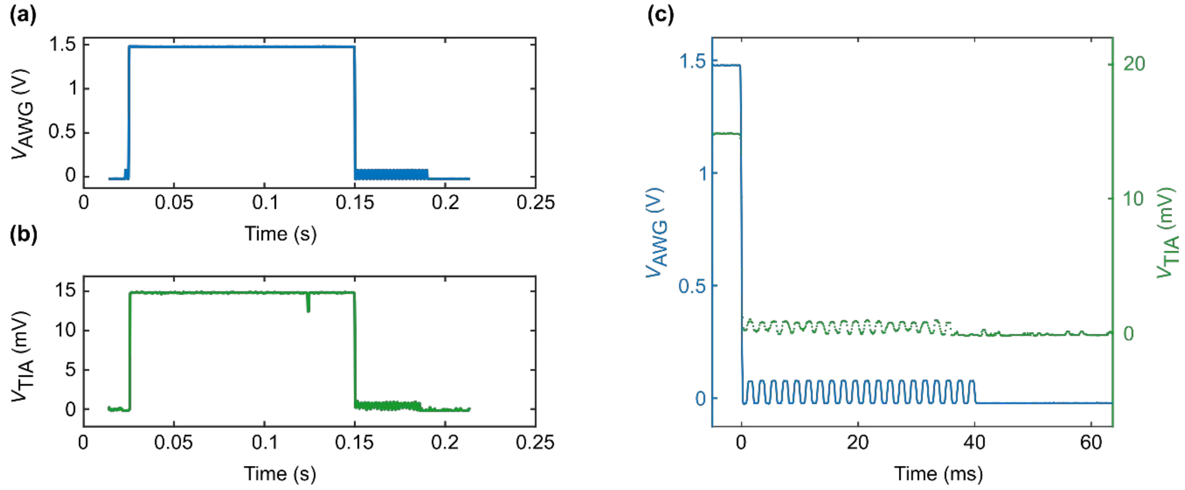

Figure S4: Pulsed retention measurements using the same bias voltage $V_B = 1.5\ V$ and series resistance $R_S = 1\ M\Omega$ as in the pulsed measurements shown in Figure 4 of the manuscript. (a) The bias voltage supplied by the AWG is shown. The read pulses have an amplitude of $0.1\ V$ and duration of $1\ ms$. After a first read pulse, a $125\ ms$ long set pulse and 20 read pulses with $1\ ms$ wait time in between follow. (b) We amplify the current after the device with a TIA and measure the output voltage. (c) End of the set pulse and the twenty read pulses are shown in detail. The AWG voltage (blue) and the measured TIA voltage (green) show that the device turns off after about $37\ ms$.

4. Full I-V Cyclic Voltammetry

The full data shown in Figure 1 and 2 of the manuscript is reproduced here. The current is plotted in logarithmic scale. All cycles are plotted in a light color, while the median cycle is highlighted in a bold color.

Certain cycles exhibit currents above 10 nA for negative source-drain voltages V_{SD}. However, this observation alone does not imply nonvolatility, especially when considered alongside the short retention times depicted in Figure S4 of the Supporting Information "Retention" and the data presented in "Reset Voltage Analysis" in the Supporting Information.

The spread in the observed onset of the device is in the typical range for Ag-based systems. [2–4]

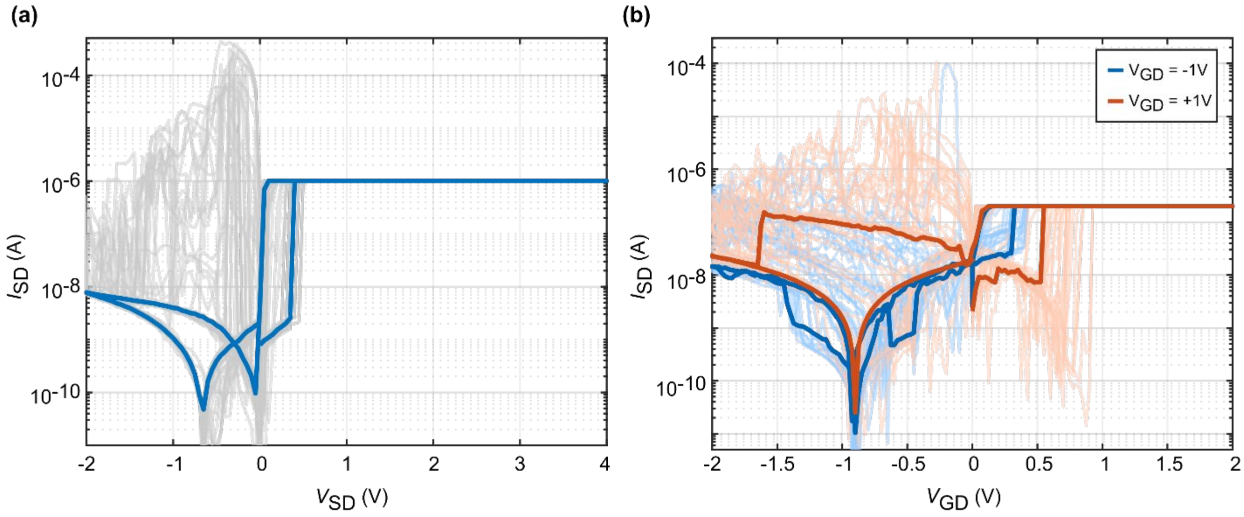

Figure S5: Full IV sweeps of the measurements shown in Figure 1 and 2 of the main text. All cycles are shown in light color, while the median cycle is highlighted. (a) Two terminal IV sweeps with a floating gate. (b) Three terminal IV sweeps with gate voltage (-1 V, +1 V) shown in blue and orange, respectively.

5. Reset Voltage Analysis

The reset voltage was extracted from the measurements shown in Figure 2 and Figure S5(b). It was evaluated as the voltage, where the device resistance $R_{\mathrm{DUT}} > 10R_{\mathrm{on}}$ with R_{on} being the mean of the resistances at V_{set} and the next 9 data points. A histogram of the found reset voltages V_{reset} is shown in Figure S6 and the statistical data is presented in Table S1. The low reset voltages fit well with the observed low retention.

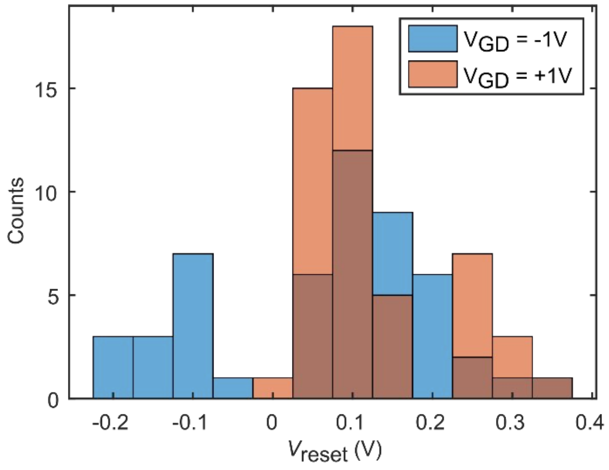

Figure S6: Reset voltage evaluation of the gate biased measurements shown in Figure 2 and Figure S5(b).The reset voltage is extracted at the point where $R_{DUT} > 10R_{on}$.

**Applied Gate Voltage**	$\boldsymbol{V_{\mathrm{G}} = +1\ \mathrm{V}}$	$\boldsymbol{V_{\mathrm{G}} = -1\ \mathrm{V}}$
$\boldsymbol{V_{\mathrm{reset}}}$ **Mean**	0.1431 V	0.0755 V
$\boldsymbol{V_{\mathrm{reset}}}$ **Median**	0.1250 V	0.1250 V
$\boldsymbol{V_{\mathrm{reset}}}$ **Standard Deviation**	0.1250 V	0.0954 V

Table S1: Statistical data evaluation of the presented reset voltages.

6. Continuous Gate Change Measurements

Using the IV measurement setup used in Figures 1 and 2, we apply a triangular voltage signal on the gate (V_{GD}), while keeping the source-drain voltage constant V_{SD} and the drain grounded, as shown in Figure S7(a) in purple and blue respectively. We perform this sweep two times and find that the gate voltage can turn the device on and off in a controllable way. In Figure S7(b), the current response I_{SD} of the source-drain contact is plotted against time. A compliance current of $I_{\mathrm{cc}} = 100\ \mathrm{nA}$ was set. In Figure S7(c), the same data is plotted against the gate voltage showing an onset for negative gate voltages.

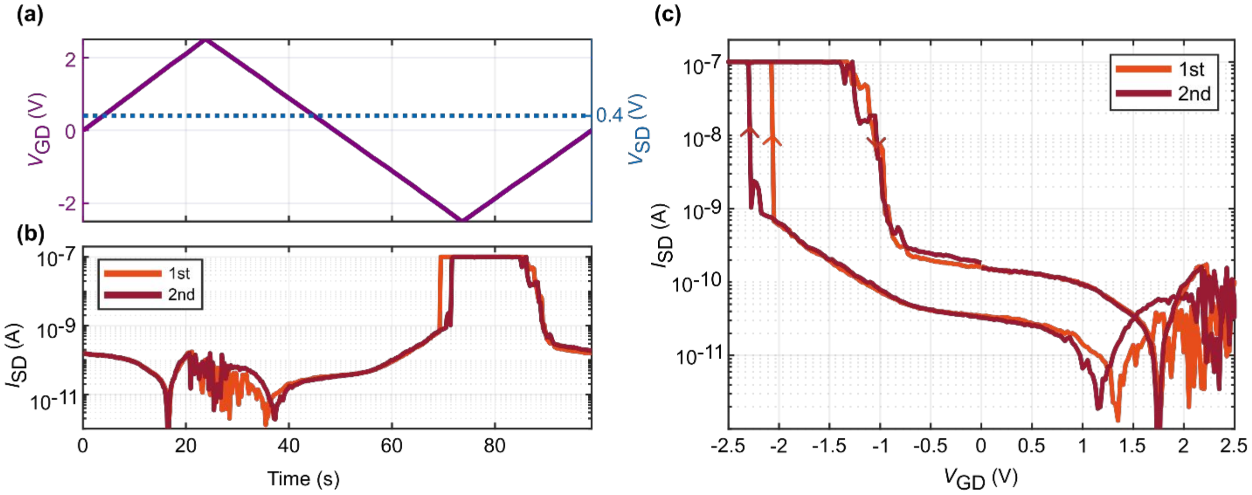

Figure S7: Continuous gate voltage sweep with the SD voltage kept constant. (a) In purple, the triangular gate voltage signal is shown ($V_{\mathrm{GD}} \in [-2.5\,V, +2.5\,V]$). In blue, the constant source-drain voltage is shown ($V_{\mathrm{SD}} = 0.4\,V$). (b) Overlay of the source-drain currents I_{SD} of two sweeps over time. One can observe a turn on in both cycles at around 70 s. (c) I_{SD} is plotted as a function of V_{GD}. We observe that negative gate voltages can turn the device on.

7. Leakage Currents

Leakage currents between gate and source or drain electrodes have been measured. To this end, we applied the voltages as used in our experiments for $2\ \mathrm{s}$, thereby gathering seven data points with an integration time of $40\ \mathrm{ms}$ for both I_{SG} and I_{DG}. We repeated these measurements on three devices. The mean current of all data points is shown in Table S2 below. Hence, leakage currents to the gate are significantly below source-drain current in compliance ($I_{\mathrm{SG}}, I_{\mathrm{DG}} \ll 100\ \mathrm{nA} \leq I_{\mathrm{SD,cc}}$).

**Applied Voltage**	$\boldsymbol{I_{\mathrm{SG}}}$ $(\mathbf{nA})$	$\boldsymbol{I_{\mathrm{DG}}}$ $(\mathbf{nA})$
$\mathbf{+1\ V}$	0.14	0.14
$\mathbf{-1\ V}$	-0.26	-0.30
$\mathbf{+2\ V}$	0.65	0.91
$\mathbf{-2\ V}$	-1.3	-1.6

Table S2: Leakage current data. In the left column, the applied voltage between the corresponding contacts is shown. All currents are below or equal to 1.6 nA, which is considerably less than the source-drain currents I_{SD} *in the gated measurements.*

8. Gate Influence on the Resistance State Retention

Gate influence on the resistance state's retention was investigated using the same setup as in Figure 4-6 of the manuscript. The bias voltage V_B was held constant while a rectangular voltage signal was applied to the gate (V_GD). After several pulses, the gate was set to floating, leading to a disconnected V_GD, as illustrated in Figure S8(a), where the transition from solid to dashed purple line indicates this event. Throughout this process, V_B remains unchanged and is shown in blue. The measured source-drain voltage V_SD, shown in Figure S8(b), was used to calculate the device resistance R_DUT, depicted in Figure S8(c).

We evaluate the retention time (τ) as the time between the end of the last gate pulse and the subsequent drop of R_DUT. The resistance drops by > 50 % in $\tau = 100$ µs when no gate signal is applied, as shown in Figure S8(d). To derive this, three resistance states are extracted from the data: the mean resistance $R_{\mathrm{VG}+}$ when applying a positive gate bias, the resistance R_float with floating gate, and the midpoint R_half of these two values. The gated retention time τ is defined as the time difference between the last point with the connected gate and the first point after the resistance has dropped below R_half.

This time scale agrees with the RC-time of the used setup, as discussed in the Method Section.

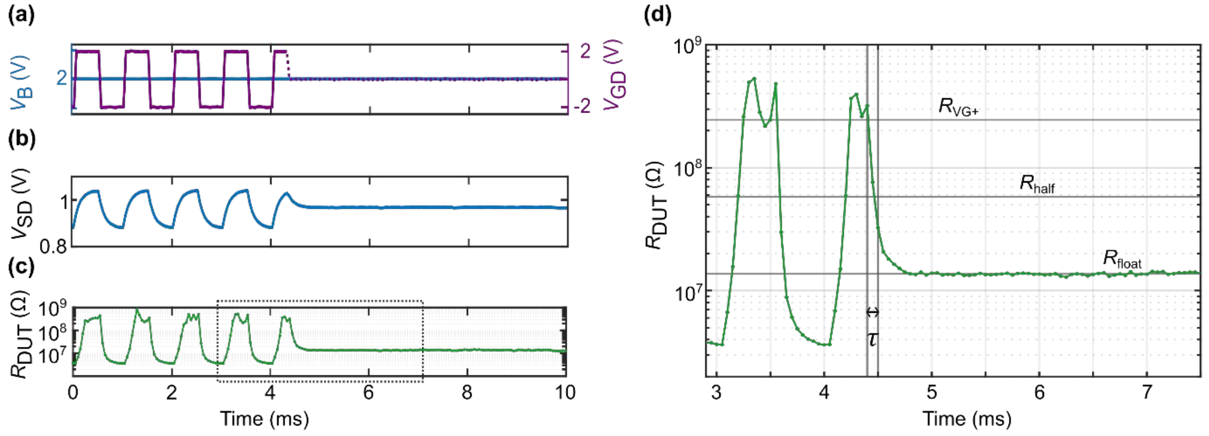

Figure S8: Resistance state retention when the gate is set to floating. (a) The bias voltage V_B, in blue, is held constant whereas the gate voltage V_{GD}, in purple, is first pulsed and then disconnected. This is depicted by the transition from a solid to a dashed line. (b) The source-drain voltage V_{SD} is measured and used to calculate the device's resistance state. (c) The calculated device resistance R_{DUT}. During the rectangular gate pulses, the device resistance switched between two states. Once the gate contact is disconnected, the resistance relaxes to an intermediate state. (d) Zoom-in to the retention measurement. The time between the gate is disconnected and the resistance is falling below R_half is extracted as the gated retention time $\tau = 100$ µs.

References

(1) Lübben, M.; Menzel, S.; Park, S. G.; Yang, M.; Waser, R.; Valov, I. SET Kinetics of Electrochemical Metallization Cells: Influence of Counter-Electrodes in SiO2/Ag Based Systems. *Nanotechnology* **2017**, *28* (13), 135205. https://doi.org/10.1088/1361-6528/aa5e59.
(2) Zhang, X.; Wu, C.; Lv, Y.; Zhang, Y.; Liu, W. High-Performance Flexible Polymer Memristor Based on Stable Filamentary Switching. *Nano Lett.* **2022**, *22* (17), 7246–7253. https://doi.org/10.1021/acs.nanolett.2c02765.
(3) Passerini, E.; Lewerenz, M.; Csontos, M.; Jimenez Olalla, N.; Keller, K.; Aeschlimann, J.; Xie, F.; Emboras, A.; Zhang, X.; Fischer, M.; Fedoryshyn, Y.; Luisier, M.; Schimmel, T.; Koch, U.; Leuthold, J. Controlling Volatility and Nonvolatility of Memristive Devices by Sn Alloying. *ACS Appl. Electron. Mater.* **2023**, *5* (12), 6842–6849. https://doi.org/10.1021/acsaelm.3c01275.
(4) Wang, Z.; Joshi, S.; Savel’ev, S. E.; Jiang, H.; Midya, R.; Lin, P.; Hu, M.; Ge, N.; Strachan, J. P.; Li, Z.; Wu, Q.; Barnell, M.; Li, G.-L.; Xin, H. L.; Williams, R. S.; Xia, Q.; Yang, J. J. Memristors with Diffusive Dynamics as Synaptic Emulators for Neuromorphic Computing. *Nat. Mater.* **2017**, *16* (1), 101–108. https://doi.org/10.1038/nmat4756.
